# Supplementary material for: Machine learning for improved density functional theory thermodynamics
Source: Sci Rep. 2025 May 17;15:17212. doi: 10.1038/s41598-025-02088-7 (PMC12085584; doi:10.1038/s41598-025-02088-7)
Supplement: Supplementary file 1 — Supplementary Information. [file 41598_2025_2088_MOESM1_ESM.pdf]

Machine Learning for Improved Density Functional Theory Thermodynamics - Supporting information

Sergei I. Simak[a,b], Erna K. Delczeg-Czirjak[b,c] and Olle Eriksson[b,c]

[a]*Department of Physics, Chemistry and Biology (IFM), Linköping University, SE-581 83 Linköping, Sweden*
[b]*Department of Physics and Astronomy, Uppsala University, Box 516, SE-75120, Uppsala, Sweden*
[c]*WISE - Wallenberg Initiative Materials Science for Sustainability, Department of Physics and Astronomy, Uppsala University, SE-751 20 Uppsala, Sweden*

ARTICLE INFO

Keywords:
Density Functional Theory (DFT), Machine Learning Corrections, Neural Network Regression, Formation Enthalpy Prediction, Phase Stability, Multicomponent Alloys, First-Principles Calculations, High-Temperature Materials

ABSTRACT

The predictive accuracy of density functional theory (DFT) for alloy formation enthalpies is often limited by intrinsic energy resolution errors, particularly in ternary phase stability calculations. In this work, we present a machine learning (ML) approach to systematically correct these errors, improving the reliability of first-principles predictions. A neural network model has been trained to predict the discrepancy between DFT-calculated and experimentally measured enthalpies for binary and ternary alloys and compounds. The model utilizes a structured feature set comprising elemental concentrations, atomic numbers, and interaction terms to capture key chemical and structural effects. By applying supervised learning and rigorous data curation we ensure a robust and physically meaningful correction. The model is implemented as a multi-layer perceptron (MLP) regressor with three hidden layers, optimized through leave-one-out cross-validation (LOOCV) and k-fold cross-validation to prevent overfitting. We illustrate the effectiveness of this method by applying it to the Al-Ni-Pd and Al-Ni-Ti systems, which are of interest for high-temperature applications in aerospace and protective coatings.

Supporting Information

The experimental heat of formation (H_{expt}) together with the predicted values from the ML model (H_{pred}) are listed in Table 1 for all intermetallic compounds and alloys investigated. In the last two columns of the table, the deviation between the predicted and measured enthalpies of formation ($H_{\text{pred}} - H_{\text{expt}}$) and the deviation between the DFT estimated and experimentally measured data ($H_{\text{DFT}} - H_{\text{expt}}$) are also listed.

References

[1] G. Kim, S. V. Meschel, P. Nash, W. Chen, Experimental formation enthalpies for intermetallic phases and other inorganic compounds, Scientific Data 4 (2017) 170162. doi:10.1038/sdata.2017.162.
URL https://doi.org/10.1038/sdata.2017.162

[2] P. Nash, O. Kleppa, Composition dependence of the enthalpies of formation of nial, Journal of Alloys and Compounds 321 (2) (2001) 228–231, special issue dedicated to Professor Ole J. Kleppa. doi:https://doi.org/10.1016/S0925-8388(01)00952-5.
URL https://www.sciencedirect.com/science/article/pii/S0925838801009525

[3] R. Hu, P. Nash, Q. Chen, Enthalpy of formation in the al-ni-ti system, Journal of Phase Equilibria and Diffusion 30 (5) (2009) 559–563. doi:10.1007/s11669-009-9573-3.
URL https://doi.org/10.1007/s11669-009-9573-3

[4] Q. Guo, O. Kleppa, Standard enthalpies of formation of some alloys formed between group iv elements and group viii elements, determined by high-temperature direct synthesis calorimetry: Ii. alloys of (ti, zr, hf) with (co, ni), Journal of Alloys and Compounds 269 (1) (1998) 181–186. doi:https://doi.org/10.1016/S0925-8388(98)00246-1.
URL https://www.sciencedirect.com/science/article/pii/S0925838898002461

ORCID(s): 0000-0002-1320-389X (S.I. Simak); 0000-0002-1667-2894 (E.K. Delczeg-Czirjak); 0000-0001-5111-1374 (O. Eriksson)

Table 1
Number of valence electrons ($n_{\text{v.e.}}$) for the investigated alloys and compounds (Composition) at their experimentally reported structures indicated by their Space Group. Measured heat of formation (H_{expt}), predicted heat of formation (H_{pred}), the deviation between the predicted and measured enthalpies of formation ($H_{\text{pred}} - H_{\text{expt}}$) and the deviation between the DFT estimated and measured data ($H_{\text{DFT}} - H_{\text{expt}}$). All energies are in units of eV/atom. If not stated otherwise, the experimental heat of formation is from the database of Ref. [1].

nr	$n_{v.e.}$	Composition	Space Group	H_{expt}	H_{pred}	$H_{\text{pred}} - H_{\text{expt}}$	$H_{\text{DFT}} - H_{\text{expt}}$
1	3.25	$Ti_{0.25}Al_{0.75}$	I4/mmm	-0.3793	-0.3791	0.0002	-0.0153
2	6.08	$Al_{0.56}Ni_{0.44}$	Pm-3m	-0.6022^a	-0.6021	0.0001	0.0450
3	6.47	$Al_{0.505}Ni_{0.495}$	Pm-3m	-0.6291^a	-0.6309	-0.0018	-0.0392
4	6.50	$Al_{0.50}Ni_{0.20}Pd_{0.30}$	Pm-3m	-0.8080	-0.8088	-0.0008	0.0444
5	6.50	$Al_{0.50}Pd_{0.50}$	Pm-3m	-0.9457	-0.9455	0.0002	0.0440
6	6.50	$Al_{0.50}Ni_{0.30}Pd_{0.20}$	Pm-3m	-0.7476	-0.7469	0.0007	0.0324
7	6.50	$Al_{0.50}Ni_{0.10}Pd_{0.40}$	Pm-3m	-0.9033	-0.9031	0.0002	0.0766
8	6.50	$Al_{0.50}Ni_{0.40}Pd_{0.10}$	Pm-3m	-0.6777	-0.6778	-0.0001	-0.0072
9	6.54	$Al_{0.495}Ni_{0.505}$	Pm-3m	-0.6343^a	-0.6319	0.0024	-0.0385
10	6.55	$Al_{0.45}Ni_{0.50}Ti_{0.05}$	Pm-3m	-0.6156	-0.6181	-0.0025	-0.0378
11	6.60	$Al_{0.40}Ni_{0.50}Ti_{0.10}$	Pm-3m	-0.6229	-0.6228	0.0001	-0.0041
12	6.70	$Ti_{0.20}Al_{0.30}Ni_{0.50}$	Fm-3m	-0.5599	-0.5598	0.0001	-0.0744
13	6.80	$Ti_{0.30}Al_{0.20}Ni_{0.50}$	Fm-3m	-0.5690	-0.5689	0.0001	-0.0002
14	6.90	$Al_{0.40}Ni_{0.55}Ti_{0.05}$	Pm-3m	-0.5882	-0.5851	0.0031	-0.0175
15	6.92	$Al_{0.44}Ni_{0.56}$	Pm-3m	-0.5835^a	-0.5753	0.0082	-0.0364
16	6.95	$Al_{0.05}Ni_{0.50}Ti_{0.45}$	Pm-3m	-0.4218	-0.4218	0.0000	0.0150
17	6.99	$Al_{0.43}Ni_{0.57}$	Pm-3m	-0.5566^a	-0.5663	-0.0097	-0.0530
18	7.00	$Ti_{0.50}Ni_{0.50}$	Pm-3m	-0.3749	-0.3747	0.0002	0.0028
19	7.27	$Al_{0.39}Ni_{0.61}$	Pm-3m	-0.5317^a	-0.5313	0.0004	-0.0337
20	7.67	$Al_{0.33}Pd_{0.66}$	Pnma	-0.9048	-0.9048	0.0000	0.0993
21	8.28	$Al_{0.16}Ni_{0.74}Ti_{0.10}$	Pm-3m	-0.4404^b	-0.4406	-0.0002	0.0107
22	8.33	$Al_{0.11}Ni_{0.74}Ti_{0.15}$	Pm-3m	-0.4102	-0.4099	0.0003	-0.0238
23	8.45	$Al_{0.05}Ni_{0.75}Ti_{0.20}$	P63/mmc	-0.3616	-0.3616	0.0000	-0.0674
24	8.50	$Ti_{0.25}Pd_{0.75}$	P63/mmc	-0.6737	-0.6737	0.0000	0.0626
25	8.50	$Ti_{0.25}Ni_{0.75}$	P63/mmc	-0.4374^c	-0.4371	0.0003	0.0016
26	6.22	$Al_{0.54}Ni_{0.46}$	Pm-3m	-0.6177	-0.6124	0.0053	0.0187
27	6.36	$Al_{0.52}Ni_{0.48}$	Pm-3m	-0.6301^a	-0.6206	0.0095	-0.0090
28	6.50	$Al_{0.50}Ni_{0.50}$	Pm-3m	-0.6405^a	-0.6384	0.0021	-0.0372
29	6.75	$Ti_{0.25}Al_{0.25}Ni_{0.50}$	Fm-3m	-0.5783	-0.5892	-0.0109	-0.0598
30	6.85	$Al_{0.45}Ni_{0.55}$	Pm-3m	-0.6053	-0.5843	0.0210	-0.0246
31	7.00	$Ti_{0.50}Pd_{0.50}$	Pmma	-0.5524	-0.5505	0.0019	0.0299
32	7.20	$Al_{0.40}Ni_{0.60}$	Pm-3m	-0.5317	-0.5401	-0.0084	-0.0451
33	8.23	$Al_{0.21}Ni_{0.74}Ti_{0.05}$	Pm-3m	-0.4456^c	-0.4385	0.0071	0.0228
34	8.25	$Al_{0.25}Ni_{0.75}$	Pm-3m	-0.4198	-0.4134	0.0064	0.0065

^{a}Ref. [2] ^{b}Ref. [3] ^{c}Ref. [4]
